# Serological Evidence of Highly Pathogenic Avian Influenza (H5N1) in Invasive Wild Pigs in Western Canada

**DOI:** 10.1155/tbed/2720469

**Published:** 2025-11-17

**Authors:** Oshin Ley Garcia, Tamiru Alkie, Frank van der Meer, Yohannes Berhane, Susan E. Detmer, Ishara Muhammadu Isham, Hannah McKenzie, Chunu Mainali, Mathieu Pruvot

**Affiliations:** ^1^Faculty of Veterinary Medicine, University of Calgary, Calgary, AB, T2N 4N1, Canada; ^2^National Centre for Foreign Animal Disease (NCFAD), Canadian Food Inspection Agency (CFIA), Winnipeg, MB, R3E 3M4, Canada; ^3^Department of Veterinary Pathology, University of Saskatchewan, Saskatoon, SK, S7N 5B4, Canada; ^4^Government of Alberta, Edmonton, AB, T5K 1E7, Canada; ^5^Wildlife Conservation Society, Health Program, Bronx, NY, USA

## Abstract

Influenza A virus (IAV) can infect a wide range of hosts, including wild and domestic pigs. Swine play an important role in influenza evolution and epidemiology due to their ability to get infected with both avian and human influenza viruses, potentially leading to reassorted virus variants. Interactions at the wild-domestic swine interface have been documented on multiple occasions, raising concern about pathogen transmission and the emergence of novel influenza strains. This study investigates the occurrence and subtypes of IAV infecting invasive wild pigs in Alberta, Canada. A total of 267 wild pigs were captured between 2021–2024. Exposure to IAV was initially detected by cELISA, with further confirmation of exposure to the H5Nx virus by hemagglutination inhibition (HI) and virus neutralization (VN) assays. Although no IAV genetic material was detected by qPCR, the seropositive samples by cELISA (4.17%; 5/120) coincided with the 2022–2024 highly pathogenic avian influenza virus (HPAI) H5N1 epizootic in Alberta, which involved outbreaks in wild species and domestic birds. These findings, combined with the epidemiological context, suggest interspecies transmission of HPAI H5N1 clade 2.3.4.4b to wild pigs. These results highlight the potential role of wild pigs as a new host in Canada and emphasize the need for continued surveillance of IAV in wild pig populations to assess the risk of spillover events at the wildlife, livestock, and human interfaces.

## 1. Introduction

Influenza A virus (IAV), a member of the *Orthomyxoviridae* family of RNA viruses [1], infects a diverse range of hosts, including domestic and wild avian, porcine, equine, canine, bovine, and feline species, as well as humans [2]. The primary natural reservoir of most subtypes of IAV is wild water birds, particularly from the orders *Anseriformes* (ducks, geese, and swans) and *Charadriiformes* (waders and gulls) [3]. The fragmented genome of IAV and its potential for reassortment with viruses originating from different host species have led to significant zoonotic spillover events. Most notably, the 2009 H1N1 influenza pandemic which emerged from a novel reassortant of three major swine lineages, resulted in widespread human infections and fatalities [4, 5]. Several distinct lineages of swine influenza virus (IAV-S), primarily subtypes H1N1, H1N2, and H3N2, have become endemic in swine populations worldwide [6, 7]. Swine are considered a key mixing vessel for IAV due to the presence of two cell surface receptors on respiratory epithelial cells (sialic acid α2,3 and α2,6) for both avian and human IAV [8].

In the Canadian prairies, wild pigs (*Sus scrofa*) have rapidly expanded their geographic range. [9], posing a serious threat to agricultural and natural ecosystems [10]. Free-ranging wild pigs are hosts to various pathogens, with potential impacts on livestock, wildlife, and human health [11, 12]. Interactions between wild and domestic pigs have been documented, especially when domestic pigs are raised outdoors or under free-ranging conditions [13]. Such interactions increase the risk of transmitting swine diseases at the wild-domestic pig interface, including brucellosis, African swine fever (ASF), and Aujeszky's disease [14–16].

IAV can serve as a valuable model to understand spillover events between wild and domestic swine and to assess transmission dynamics [17]. IAV infection has been described in wild pig populations across multiple regions, including Poland and Spain [18], Croatia [19], Italy [20, 21], and Germany [22]. In the United States, feral swine (*Sus scrofa*) have been exposed to both swine and avian influenza, with a higher prevalence of IAV-S [23]. Genetic reassortment and the emergence of novel IAV strains have been reported in interactions of domestic and feral swine in the southeastern and south-central United States [24], highlighting the role of swine as “mixing vessels” for influenza virus of multiple origin, and a potential driver in the reassortment and evolution of new strains.

Recently, the emergence of highly pathogenic avian influenza virus (HPAI) H5N1, characterized by hemagglutinin (HA) clade 2.3.4.4b, descended from the H5N8 lineage that previously dominated across Eurasia [25, 26], has become a major health concern in North America, due to frequent spillover between wild and domestic birds, and into a variety of mammalian species [27]. In 2021, this virus was identified in poultry and seabirds in Eastern Canada [28]. Since then, HPAI cases have been detected across 12 taxonomic orders of wild birds and 80 species in all Canadian provinces and territories, with the highest numbers reported in the Atlantic and Central Flyways [29]. In 2022, an unusual mortality event involving seals in Quebec, Canada, was linked to HPAI H5N1 infection, likely resulting from exposure to infected wild bird carcasses and subsequent spillover to marine mammals [30]. Similar cases were reported in seals from New England and Washington, United States, as well as Argentina, where HPAI H5N1 clade 2.3.4.4b was confirmed [31–33]. In South America, HPAI-H5N1 has been detected in marine mammals, including seals, sea lions, dolphins, otters, and penguins, across Peru, Chile, Brazil, and Uruguay [34].

More recently, outbreaks in dairy cattle across the United States have further underscored the risk posed by HPAI H5N1 [35]. The continued spread of this virus highlights the ongoing threat to animal health, public health, and conservation [36].

While extensive research has documented the circulation of IAV in Canadian domestic pigs, including subtypes H1N1, H1N2, H3N2 human-swine reassortant, and H4N6 [37–41], no study so far has investigated the occurrence of IAV in invasive wild pig populations to understand transmission at the wild-domestic swine interface. Notably, during the 2023–2024 surveillance period, only two cases of H1N1 1.A.1.1 (Alpha) were reported in domestic pigs in Alberta, and no H3N2 were detected [42]. This study aims to provide an overview of the occurrence and subtypes (including HPAI H5N1) of IAV circulating in the invasive wild pig population of Alberta, Canada.

We hypothesize that interactions with wild birds and domestic pigs will result in exposure of both avian and swine IAV in wild pigs.

## 2. Materials and Methods

### 2.1. Study Design

As part of an opportunistic surveillance effort supported by the Alberta Wild Boar Control Program, 267 individual animals were captured and euthanized between 2021–2024, in the counties of Woodland, Lac Ste. Anne, Two Hills, and Strathcona in the province of Alberta, Canada. Shortly after euthanasia, 3 mL of blood was collected directly from the heart into a sterile BD Vacutainer tube (ThermoFisher Scientific, Waltham MA, USA). After euthanasia, carcasses were transported to the Agri-Food Assurance Section, Food Safety Branch, Trade Investment & Food Safety Division, Alberta Agriculture and Irrigation (Edmonton, Alberta) for necropsy. In total, 267 lung samples were collected, and 120 serum samples were stored at −80°C for subsequent serological analyses. Wild pigs were categorized by sex (male and female) and age (juvenile and adult), with juveniles identified by the presence of stripes and adults by their absence.

### 2.2. Serology

#### 2.2.1. ELISA

Serum samples were first evaluated for the presence of antibodies against IAV using a nucleoprotein (NP) specific competitive ELISA (Biovet, St. Hyacinthe, QC, Canada).

Reactive sera (seropositive samples by NP cELISA) and regionally significant virus field isolates from Canada were initially sent to the University of Minnesota Veterinary Diagnostic Laboratory (UMN-VDL, St. Paul, Minnesota, USA) and subsequently to the National Centre for Foreign Animal Disease (NCFAD, Winnipeg, Manitoba, Canada) for IAV subtype determinations.

#### 2.2.2. Hemagglutination Inhibition (HI)

HI assays were tested according to the standard protocol routinely performed at UMN-VDL against selected reference antigens of IAV from swine (IAV-S), as detailed in Table S1. These field isolates were genetically similar to the viruses circulating in Alberta at the time that the serum samples were collected, including: 2009pandemicH1N1 or 1A.3.2.2 clade (A/swine/BC/SD0391/2019); Alpha-3 H1N2 or 1.A.1.1 clade (A/swine/AB/SD0191/2016 [MF768475]; A/swine/AB/SD0545/2020; A/swine/MB/56317/2022; A/swine/AB/SD0948/2023), and H3N2 clade IV or 1990.4 (A/swine/AB/SD0622/2021 IVE; A/swine/AB/SD0659/2021 IVC1; A/swine/MB/58116/2022 IVB2). Subsequently the sera were tested with an avian influenza H5N9 virus-like particles (VLPs) containing clade 2.3.4.4b HA at NCFAD. The H5N9 VLPs were developed at NCFAD using the baculovirus–insect cell expression system, incorporating a consensus HA gene based on clade 2.3.4.4b H5N1 influenza viruses reflecting the predominant HA found in Canadian recent outbreaks. The neuraminidase (NA) segment corresponds to a North American lineage N9 viruses. The resulting H5N9 VLPs demonstrated strong hemagglutination activity and were validated at NCFAD for use in HI assays.

Briefly, serum samples (100 μL) were treated with 4 volumes of a receptor-destroying enzyme (RDE) (Denka Seiken, Tokyo, Japan), followed by heat inactivation at 56°C, and subsequently adsorbed with chicken red blood cells (RBCs). The treated serum was two-fold serially diluted, and an equal volume containing four hemagglutination units of H5N9 (carrying clade 2.3.4.4b HA) VLPs was added as an antigen in the HI assay. HI antibody titers were determined as the reciprocal of the highest serum dilution resulting in complete inhibition of hemagglutination of the chicken RBCs and expressed as log_2_ titers. The resulting HI titers were adjusted to account for the dilution of serum samples with RDE. Each sample was run in duplicate, and H5-positive control sera were included in every run to confirm assay performance and exclude false negatives.

#### 2.2.3. Virus Neutralization (VN) Test

HI-positive sera were tested for H5-specific neutralizing antibodies using the VN Test (VNT). Briefly, MDCK cells (CCL-34; ATCC) were seeded at a density of 1.5 × 10^4^/well in 96-well tissue culture plates (Corning) in Dulbecco's Modified Eagle's Medium (DMEM, Sigma, St Louis, MO, USA) supplemented with 10% fetal bovine serum (FBS) and 1× GlutaMAX (ThermoFisher Scientific, Waltham MA, USA) and 100 U/mL of penicillin-streptomycin. Heat-inactivated serum was diluted at 1/10 with DMEM media. A two-fold serial dilution of the serum/DMEM was mixed with 100 TCID50 of H5N1 (clade 2.3.4.4b) HPAI (A/Qc/Dk/FAV128-1/2023) (GISAID EPI_ISL_19155247) and incubated at 37°C for 1 h. The virus–serum mixture was added to the MDCK-monolayer and incubated at 37°C in a 5% CO_2_ humidified incubator. Each sample was run in duplicate, with virus- and cell-based positive controls included in each run to confirm assay integrity and detect any potential assay failure. Virus-neutralizing titers were defined as the highest serum dilution that blocked the occurrence of CPE.

Samples were considered positive for prior IAV exposure when either the HI or VNT titer was ≥40 [43], using this threshold as a conservative indicator in the absence of validated cutoffs for swine. All VNT procedures were conducted in a biosafety level 3 (BSL-3) facility at the NCFAD.

### 2.3. Molecular Screening

A subset of lung samples was selected for IAV detection, prioritizing (i) samples collected during periods of HPAI H5N1 outbreak activity, (ii) groups located in geographical proximity to other wild pig sounders, and (iii) groups with serological evidence of prior exposure. Total RNA was extracted from 10 mg of lung samples using TRIzol Reagent (ThermoFisher Scientific, Waltham MA, USA) following the manufacturer's recommendations. 2000 ng of RNA was reverse transcribed into complementary DNA (cDNA) using a high-capacity cDNA reverse transcription Kit (Applied Biosystems, Waltham MA, USA) as per the manufacturer's instructions in a T100 Thermal Cycler (BIO-RAD, Hercules, CA, USA). The concentration and purity of the RNA and cDNA from all samples were determined using a NanoDrop 1000 Spectrophotometer (Thermo Scientific, Wilmington, Delaware, USA). Primers used [44] targeting the Influenza A Matrix (M) gene are described in Table S2. The qPCR reaction mixture consisted of 400 nM of each primer, 6.5 μL nuclease-free H_2_0, 4 μL (800 ng) cDNA, and 12.5 μL SYBR Green (Quntabio, Beverly MA, USA) in a 25 μL final volume. The reactions were run in a CFX96 Real-Time System (BIO-RAD) under the following conditions: 45°C for 10 min, 95°C for 10 min, followed by 40 cycles of 94°C for 5 s, and 60°C for 1 min. In vitro transcribed (IVT) RNA controls for the M gene, prepared and supplied by NCFAD, were used as positive control. Negative controls (RNase-free water) and positive controls (IVT RNA) were included in each qPCR run. Samples yielding a cycle threshold (C_t_) value below 35.99 were interpreted as positive.

### 2.4. Statistical Analysis

The seroprevalence of IAV and 95% confidence interval (CI) was estimated using the epiR package from the R environment v4.4.0. For spatial analysis, the locations of wildlife and domestic birds HPAI H5N1 cases, and wild pigs trap sites were compiled from publicly available surveillance data and field records. Wildlife HPAI H5N1 data were sourced from the Canadian Wildlife Health Cooperative Dashboard. Data for domestic birds HPAI H5N1 were obtained from the Canadian Food Inspection Agency Power BI Dashboard. The maps were projected in NAD83/Alberta 10-TM (Forest), EPSG: 3400. Spatial data preparation and mapping were completed using QGIS v3.34.12.

## 3. Results

At the time of capture, no visible signs of infection were reported by the trapping team. Over a 4-year period (2021–2024), a total of 120 wild pigs were serological screened, with 61 samples collected in 2021, 26 in 2022, 9 in 2023 and 24 in 2024. The majority of samples were collected from female (55.8%), mature individuals (80%), and wild pigs captured in Woodland County (75.8%), with most captures occurred during the spring season (45.8%) (Table S3).

Overall, 4.17% (5/120, CI 1.37%–9.46%) of wild pigs were seropositive for IAV using the commercial cELISA kit. Seropositive individuals were identified in June 2022 (*n* = 4), and February 2024 (*n* = 1). The HI assay indicated that these five seropositive wild pigs were negative for regionally significant IAV-S subtypes (H1 and H3). Additionally, the HI assay and VNT confirmed H5-specific antibodies in the cELISA positive samples, with one sample testing negative in the HI assay but positive by VNT (Table 1).

**Figure 1:**
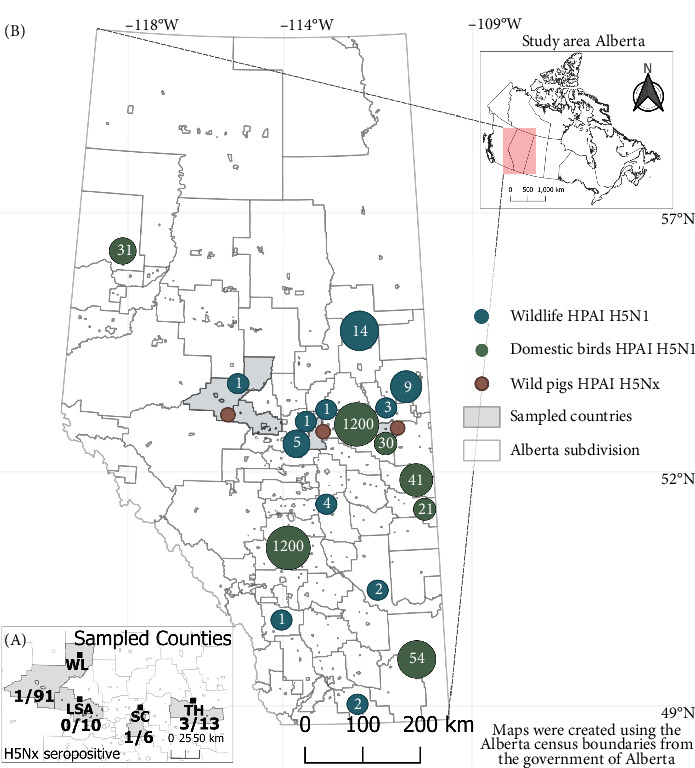
Geographical distribution of sampling sites and HPAI H5Nx seropositivity among captured wild pigs in Alberta, Canada. (A) Sampled counties from 2021–2024, including Woodland (WL), Lac Ste. Anne (LSA), Strathcona (SC), and Two Hills (TH). For each collection site, the number of seropositive samples and the proportion of tested samples are represented. (B) Confirmed cases of HPAI H5N1 in wildlife species (waterbirds, waterfowl, wild birds, striped skunk, and red foxes) between June 2022 and February 2024 are displayed with blue circles indicating the number of cases. Confirmed cases of HPAI H5N1 in domestic bird species (chickens, geese, turkeys, ducks, quails, pheasants, and partridges) between May 2022 and February 2024 are shown with green circles indicating the number of cases. Additionally, the captured location of wild pigs with HPAI H5Nx identified are displayed (red circle).

All lung samples tested with the M gene qPCR were negative for IAV genetic material (Table S4). The location of seropositive H5Nx wild pigs, along with concurrent HPAI H5N1 clade 2.3.4.4b lineages identified in wildlife species and domestic birds in Alberta, is represented in Figure 1.

## 4. Discussion

In this study, all seropositive samples coincided with the period of the 2022–2024 HPAI H5N1 epizootic in Alberta. Outbreaks of the HPAI H5N1 clade 2.3.4.4b lineage affected backyard poultry farms and commercial flocks involving layer chicken, broiler chicken, geese, and ducks [45]. Additionally, during the same timeframe, outbreaks were detected in various wild species, including waterbirds, waterfowl, other wild birds, striped skunks, and red foxes [46]. While we did not detect genetic material from the virus, the serological evidence, combined with epidemiological context, is indicative of an immune response to an infection with HPAI H5N1 clade 2.3.4.4b. Due to the transient nature of viral excretion in swine, typically characterized by a brief shedding period of approximately 5–7 days after exposure [47], it was not expected to detect genetic material of IAV. Testing for viral excretion is particularly challenging in wildlife surveillance, where virus excretion is brief and clinical signs are unobserved. Importantly, experimental infections studies with HPAI H5N1 in domestic pigs have demonstrated low susceptibility to infection, absence of severe disease, and limited viral replication, with viral RNA detected for a short duration (typically 3–5 days post-inoculation) [48–50]. These findings indicate that, while the transient shedding pattern is well-established for IAV-S, HPAI H5N1 virus exhibit even more restricted replication in pigs.

We can hypothesize several pathways through which wild pigs could encounter this virus and become infected. First, as opportunistic predators and scavengers [51], wild pigs may be exposed to the HPAI H5N1 virus by consuming infected wild bird carcasses. Similarly, spillover infections are described in free-living mammalian mesocarnivores [52]. Second, wild pigs may interact with small flocks in backyard settings, where low biosecurity measures can increase the risk of IAV transmission [53]. Finally, contaminated environments may facilitate indirect transmission of HPAI H5N1 to wild pigs through contamination of food or water sources by infected poultry or wild birds.

Natural infections of swine with HPAI H5N1 are not frequently observed. Only a limited number of studies have reported the occurrence of H5N1 in domestic pigs, with findings indicating rates of 0.18% in China [54], 0.25% in Vietnam [55], 7.4% in Indonesia [56], and 14% in Nigeria [57]. Notably, in 2021, in Rome, Italy, free-ranging pigs without clinical sign tested serologically positive for an H5N1 strain homologous to HPAI H5N1 clade 2.3.4.4b, which had previously been detected during an outbreak in neighboring backyard laying hens, although RT-PCR results in pigs were negative [58]. More recently, the first documented case of H5N1 infection in swine in the United States was reported in October 2024 on a backyard farm in Oregon, where H5N1 was initially detected in poultry before subsequently being identified in co-housed pigs [59].

This study provides evidence of antibodies against HPAI H5N1 in free-ranging invasive wild pigs in Canada; similar findings were reported in Saskatchewan (T. Bollinger, personal communication). Since these wild pigs were captured live, they clearly survived the infection. The detection of antibodies against H5N1 clade 2.3.4.4b viruses in wild pigs raises questions about the potential role of this species as a bridge host, with implications for spillover risk, inter-species transmission, and viral evolution. As wild and domestic pigs are conspecific (*Sus scrofa*), their genetic relatedness may facilitate pathogen transmission exchange [60]. This is considered particularly concerning in backyard farming systems, which often have limited or no biosecurity.

Given their susceptibility to multiple influenza virus subtypes, swine—including wild pigs—are recognized as potential mixing vessels for influenza viruses [61]. Their ability to facilitate genetic exchange among influenza viruses raises concerns about their role in viral evolution and including the emergence of new reassortants, which warrants continued monitoring given the circulation of H5N1 viruses in the Americas.

Several studies have documented IAV exposure in wild pigs, reporting seroprevalence of 1.4% in France [62], 3.4% in Japan [6], 3.5% in South Korea [63], 4.9% in the United States [64], 5.2% in Germany [65], 5.5% in Northern Italy [66], 6.4% in Spain [67], and 9.7% in Croatia [19]. The seroprevalence in our study (4.17% overall) was comparable with these reports, but notably lower than might be expected. The 2023 census data show that of 1100 farms in Alberta report pigs on premises [68]. With 278 (25.3%) farms registered in the Alberta Pork quality assurance program, most of the remaining 819 (74.5%) are smallholder and backyard farms [69]. This farm structure could increase opportunities for contact between wild and domestic pigs. However, surveillance during the study period detected only a few cases of IAV-S in domestic herds [42]. Consequently, transmission of IAV from domestic pigs to wild pigs could be low or absent. The limited detection of IAV-S in wild pigs during the period suggest that this virus may not have been the most suitable marker to assess domestic-wild transmission.

According to Peacock et al. [36], most mammalian infections of HPAI H5N1, particularly carnivores, are considered “dead-end” hosts and are not typically associated with onward transmission. The relatively low seropositivity rate observed in this study may indicate that mammal-to-mammal transmission of HPAI H5N1 among wild pigs is unlikely, although this cannot be definitively confirmed from our data. Although three of the five seropositive wild pigs originated from the same group, this does not constitute evidence of within-group transmission; however, it remains an important observation to report. Instead, the detected seroconversion in wild pigs more likely reflects sporadic spillover events from infected avian sources or shared environmental exposure during the 2022–2024 epizootic in Alberta.

Our findings may also be influenced by the sampling strategy. The Alberta Wild Boar Control Program employs whole-sounder trapping, focused on specific areas with suspected high wild pig densities. Given the extended process involved in conditioning wild pig groups for trapping, only a limited number of groups were removed during the IAV outbreak period, and sampling was not evenly distributed across the entire range of wild pigs. Additionally, this method likely excludes sick or debilitated individuals, as they may be less likely to approach traps. Notably, no dead wild pigs have been found or sampled. This could limit our ability to capture the extent of outbreaks or the frequency of spillover events into the wild pig population. Serum samples (*n* = 120) were only available from the period 2021–2024. Blood collection was performed opportunistically during field necropsy, and due to logistic constraints, serum was obtained from only 120 of the 267 wild pigs. To improve future surveillance efforts alternative sampling approaches-such as opportunistic sampling of found-dead individuals, or collaboration with hunters should be considered to enhance detection sensitivity of IAV circulation. Furthermore, the limited number of seropositive samples did not allow for the assessment of the effect of risk factors, such as age, sex, and counties, on the presence of IAV.

Despite these limitations, this study underscores the importance of ongoing surveillance to clarify the role of wild pigs in IAV maintenance and transmission. Future research should focus on genomic characterization of influenza viruses circulating in wild, domestic pigs, and avian hosts, to refine our understanding of cross-species transmission risks.

## 5. Conclusion

In summary, this study provides evidence of exposure of wild pigs to HPAI H5N1, likely originating from the concurrent epizootic in wild birds, domestic poultry, and other wildlife species, suggesting interspecies transmission of HPAI H5N1 viruses. Although serological evidence of IAV infection was observed at low prevalence in the wild pig population in Alberta, these findings raise important questions about the potential role of wild pigs in the ecology of HPAI H5N1 viruses in Canada and across North America. In the context of recurring outbreaks of H5N1, the possibility that invasive wild pigs could act as a mixing vessel and contribute to the emergence of new reassortants highlights the need for ongoing genomic surveillance and targeted research.

## Figures and Tables

**Table 1:** Animal characteristics, cELISA, HI, and VNT results of the five IAV serologically positive samples from wild pigs (*Sus scrofa*) in Alberta, Canada.

Baseline characteristics					Serological results					
					cELISA for IAV	HI titers^b^				VNT titers^c^
Sample ID	Sex	Age	County	Collection Year	(S/N)^a^	H1N1^1^	H1N2^2^	H3N2^3^	H5N9^4^	H5N1^1^
WB3	M	Mature	SC	2022	0.09	<2	<2	<2	64	1280
WB8	F	Mature	TH	2022	0.11	<2	<2	<2	64	2560
WB9	F	Mature	TH	2022	0.12	<2	<2	<2	32	640
WB10	M	Mature	TH	2022	0.24	<2	<2	<2	<2	1280
WB70	M	Mature	WL	2024	0.51	<2	<2	<2	64	1280

Abbreviations: F, Female; M, Male; SC, Strathcona; TH, Two Hills; WL, Woodland.

^a^Sample to/negative ratio under 0.600 indicates positive samples for IAV.

^b^H1N1^1^ (A/swine/BC/SD0391/2019), H1N2^2^ (A/swine/AB/SD0191/2016; A/swine/AB/SD0545/2020; A/swine/MB/56317/2022; A/swine/AB/SD0948/2023), H3N2^3^ (A/swine/AB/SD0622/2021 IVE; A/swine/AB/SD0659/2021 IVC1; A/swine/MB/58116/2022), H5N9^4^ clade 2.3.4.4b VLPs (A/FancyChicken/NL/FAV-0033/2021).

^c^H5N1^1^ clade 2.3.4.4b (A/Qc/Dk/FAV128-1/2023).

## Data Availability

The data that support the findings of this study will be available from the corresponding author (Mathieu Pruvot), upon reasonable request.

## Supporting Information

Supporting Information Table S1. Characteristic of selected IAV antigens used in hemagglutination inhibition (HI) assays and virus neutralization test (VNT). Table S2. List of sequence primers used for qPCR amplification of Influenza A Matrix (M) gene. Table S3. Frequency distribution of biological and geographical variables in wild pig serological survey (*n* = 120, 2021−2024). Table S4. Overall qPCR results for Influenza A Matrix (M) gene performed on lung samples from wild pigs (*Sus scrofa*) captured from 2022 to 2024.
