# Supplementary material for: Serological Evidence of Highly Pathogenic Avian Influenza (H5N1) in Invasive Wild Pigs in Western Canada
Source: Transbound Emerg Dis. 2025 Nov 17;2025:2720469. doi: 10.1155/tbed/2720469 (PMC12643681; doi:10.1155/tbed/2720469)
Supplement: Supporting Information — Table S1. Characteristic of selected IAV antigens used in hemagglutination inhibition (HI) assays and virus neutralization test (VNT). Table S2. List of sequence primers used for qPCR amplification of Influenza A Matrix (M) gene. Table S3. Frequency distribution of biological and geographical variables in wild pig serological survey (n = 120, 2021−2024). Table S4. Overall qPCR results for Influenza A Matrix (M) gene performed on lung samples from wild pigs (Sus scrofa) captured from 2022 to 2024. [file 2720469.f1.docx]

**SUPPLEMENTARY INFORMATION**

**TABLE S1** Characteristic of selected IAV antigens used in Hemagglutination Inhibition (HI) assays and Virus Neutralization Test (VNT).

| **Selected virus antigen** | **Source/Strain Name** | **Clade** | **Location of origin** | **Year of isolation** | **Hemagglutinin (HA) gene or Accession Number** |
| --- | --- | --- | --- | --- | --- |
| 2009pandemicH1N1 | A/swine/BC/SD0391/2019 H1N1 | 1A.3.2.2 | British Columbia | 2019 | [GenBank: PX393443] |
| Alpha-3 or AlphaDel | A/swine/AB/SD0191/2016 H1N2 | 1.A.1.1 | Alberta | 2016 | [GenBank: MF768475] |
| Alpha-3 or AlphaDel | A/swine/MB/56317/2022 H1N2 | 1.A.1.1 | Manitoba | 2022 | [GenBank: PX393446] |
| Alpha-3 or AlphaDel | A/swine/AB/SD0948/2023 H1N2 | 1.A.1.1 | Alberta | 2023 | [GenBank: PX393445] |
| Alpha-3a or NoDel | A/swine/AB/SD0545/2020 H1N2 | 1.A.1.1 | Alberta | 2020 | [GenBank: PX393444] |
| H3N2 IVE-like | A/swine/AB/SD0622/2021 H3N2 | 1990.4 | Alberta | 2021 | [GenBank: PX393447] |
| H3N2 IVC-like | A/swine/AB/SD0659/2021 H3N2 | 1990.4 | Alberta | 2021 | [GenBank: PX393448] |
| H3N2 IVB-like | A/swine/MB/58116/2022 H3N2 | 1990.4 | Manitoba | 2022 | [GenBank: PX393449] |
| H5N9 VLPs | A/FancyChicken/NL/FAV-0033/2021 | 2.3.4.4b | Netherlands | 2021 | [GenBank: PV312104] |
| H5N9 | A/Chicken/QC/FAV-0128-/2023 | 2.3.4.4b | Quebec | 2023 | [GISAID EPI_ISL_19155247] |

**TABLE S2** List of sequence primers used for qPCR amplification of Influenza A Matrix (M) gene.

| **Specificity** | **Gene** | **Primer name** | **Sequence (5’-3’)** | **Amplicon size (bp)** | **Orientation** | **Temperature (**º**C)** |
| --- | --- | --- | --- | --- | --- | --- |
| Influenza A  2009 Matrix | Matrix | Inf-A 2009  matrix- For | AGA TGA GTC **Y^1^**TC TAA CCG AGG TCG | 99 | Forward | 60 |
|  |  | Inf-A 2009  matrix- Rev | TGC AAA **R^2^**AC A**Y^1^Y^1^** TTC **M^3^**AG TCT CTG |  | Reverse |  |

^1^**Y** = C or T; ^2^**R** = G or A ^3^**M** = A or C ^4^**W** = A or T

**TABLE S3** Frequency distribution of biological and geographical variables in wild pig serological survey (n=120, 2021-2024).

| **Variable** | **Categories/**  **Levels** | **Frequency (n)** | **Proportion (%)** | **95% CI** |
| --- | --- | --- | --- | --- |
| **Sex (n=120)** | Male | 53 | 44.2 | 35.11-53.52 |
|  | Female | 67 | 55.8 | 46.48-64.89 |
| **Age (n=120)** | Juvenile | 24 | 20.0 | 13.25-28.28 |
|  | Mature | 96 | 80.0 | 71.72-86.75 |
| **County of captured (n=120)** | Woodland | 91 | 75.8 | 67.17-83.18 |
|  | Lac Ste. Anne | 10 | 8.4 | 4.07-14.79 |
|  | Two Hills | 13 | 10.8 | 5.90-17.81 |
|  | Strathcona | 6 | 5.0 | 1.86-10.57 |
| **Season of captured (n=120)** | Winter | 44 | 36.7 | 28.06-45.95 |
|  | Spring | 55 | 45.8 | 36.71-55.17 |
|  | Summer | 13 | 10.8 | 5.90-17.81 |
|  | Fall | 8 | 6.7 | 2.92-12.71 |
| **Year** | **Month** |  |  |  |
| **2021 (n=61)** |  |  | **50.8** | **41.55-60.07** |
|  | January | 14 | 23.0 | 13.15-35.50 |
|  | February | 5 | 8.2 | 2.72-18.10 |
|  | March | 6 | 9.8 | 3.70-20.19 |
|  | May | 28 | 45.9 | 33.06-59.15 |
|  | October | 8 | 13.1 | 5.84-24.22 |
| **2022 (n=26)** |  |  | **21.7** | **14.67-30.11** |
|  | January | 4 | 15.5 | 4.36-34.87 |
|  | March | 1 | 3.8 | 0.10-19.64 |
|  | April | 7 | 26.9 | 11.57-47.79 |
|  | May | 1 | 3.8 | 0.10-19.64 |
|  | June | 13 | 50.0 | 29.92-70.08 |
| **2023 (n=9)** |  |  | **7.5** | **3.49-13.76** |
|  | January | 9 | 100 | 66.37-100 |
| **2024 (n=24)** |  |  | **20.0** | **13.25-28.88** |
|  | January | 12 | 50.0 | 29.11-70.89 |
|  | February | 12 | 50.0 | 29.11-70.89 |

*Serum samples (n = 120) were only available from the period 2021–2024. Blood collection was performed opportunistically during field necropsy, and due to logistic constraints, serum was obtained from only 120 of the 267 wild pigs sampled.

**TABLE S4** Overall qPCR results for Influenza A Matrix (M) gene performed on lung samples from wild pigs *(Sus scrofa)* captured from 2022 to 2024.

| **Period/County** | **Inf-A 2009 Matrix** |
| --- | --- |
|  | **Lungs (n=70)** |
|  | **No. positive/No. sample** |
| **2022** |  |
| Strathcona | 0/6 |
| Two Hill | 0/12 |
| Woodland | 0/37 |
| Lac Ste. Anne | 0/2 |
| **2023** |  |
| Strathcona | 0/1 |
| Two Hill | 0/2 |
| Woodland | 0/9 |
| **2024** |  |
| Woodland | 0/1 |
